# Simultaneous Expression of *PDH45* with *EPSPS* Gene Improves Salinity and Herbicide Tolerance in Transgenic Tobacco Plants

**DOI:** 10.3389/fpls.2017.00364

**Published:** 2017-03-24

**Authors:** Bharti Garg, Sarvajeet S. Gill, Dipul K. Biswas, Ranjan K. Sahoo, Nandkumar S. Kunchge, Renu Tuteja, Narendra Tuteja

**Affiliations:** ^1^International Centre for Genetic Engineering and BiotechnologyNew Delhi, India; ^2^Stress Physiology and Molecular Biology Lab, Centre for Biotechnology, Maharshi Dayanand UniversityRohtak, India; ^3^Bejo Sheetal Seeds Pvt. LtdJalna, India; ^4^Amity Institute of Microbial Technology, Amity University Uttar PradeshNoida, India

**Keywords:** EPSPS, PDH45, salinity, herbicide tolerance, tobacco

## Abstract

To cope with the problem of salinity- and weed-induced crop losses, a multi-stress tolerant trait is need of the hour but a combinatorial view of such traits is not yet explored. The overexpression of *PDH45* (pea DNA helicase 45) and *EPSPS* (5-enoylpruvyl shikimate-3-phosphate synthase) genes have been reported to impart salinity and herbicide tolerance. Further, the understanding of mechanism and pathways utilized by PDH45 and EPSPS for salinity and herbicide tolerance will help to improve the crops of economical importance. In the present study, we have performed a comparative analysis of salinity and herbicide tolerance to check the biochemical parameters and antioxidant status of tobacco transgenic plants. Collectively, the results showed that *PDH45* overexpressing transgenic lines display efficient tolerance to salinity stress, while *PDH45+EPSPS* transgenics showed tolerance to both the salinity and herbicide as compared to the control [wild type (WT) and vector control (VC)] plants. The activities of the components of enzymatic antioxidant machinery were observed to be higher in the transgenic plants indicating the presence of an efficient antioxidant defense system which helps to cope with the stress-induced oxidative-damages. Photosynthetic parameters also showed significant increase in *PDH45* and *PDH45+EPSPS* overexpressing transgenic plants in comparison to WT, VC and *EPSPS* transgenic plants under salinity stress. Furthermore, *PDH45* and *PDH45+EPSPS* synergistically modulate the jasmonic acid and salicylic acid mediated signaling pathways for combating salinity stress. The findings of our study suggest that pyramiding of the *PDH45* gene with *EPSPS* gene renders host plants tolerant to salinity and herbicide by enhancing the antioxidant machinery thus photosynthesis.

## Introduction

Being sessile, plants are confronted with a wide range of environmental cues on regular basis, which eventually impose serious constraints on growth and development, thus productivity. Among major abiotic stress factors, salinity and weed stress are the major environmental challenges restricting plant growth and agricultural productivity in large (Mahajan and Tuteja, 2005; Funke et al., 2006; Tuteja, 2007). At molecular level, these abiotic stresses emerge as quantitative traits, which are generally regulated by multiple genes rather than a single gene. However, literature also revealed that only single gene manipulation can also impart significant stress tolerance in plants (Cushman and Bohnert, 2000; Zhang and Blumwald, 2001; Sanan-Mishra et al., 2005; Vashisht et al., 2005; Huda et al., 2013; Tuteja et al., 2013).

The development of transgenic plants through genetic manipulations has proved promising to reduce agricultural losses due to various abiotic stress factors, which eventually resulted in improved crop productivity (Mittler et al., 2004; Gill et al., 2014). The role of DNA/RNA helicases have also emerged in salinity stress tolerance (Vashisht and Tuteja, 2006; Gill et al., 2013; Owttrim, 2013; Tuteja et al., 2013). Helicases unwind the duplex nucleic acids and are involved in multitude of vital processes including replication, repair, recombination, and transcription (Tuteja and Tuteja, 2004; Tuteja et al., 2012). In this pursuit, our laboratory has explored the over expression and characterization of some of the helicase genes such as *PDH45, PDH47, SUV3*, and *p68* toward the development of salinity tolerant tobacco and rice transgenic plants without yield penalty (Sanan-Mishra et al., 2005; Vashisht et al., 2005; Amin et al., 2012; Gill et al., 2013; Tuteja et al., 2013, 2014; Banu et al., 2015).

Besides salinity stress, weeds are also considered as one of the potent contributors in reducing the agricultural productivity by competing with the Crop plants of interest for nutrition, thus affect its growth and productivity (Délye et al., 2013). Most of the herbicides control weeds by inhibiting specific enzymes by binding at the active site of the enzyme, also target photosynthesis and lipid biosynthesis (Cole et al., 2000; Wakabayashi and Böger, 2002). Literature reveals that there are two types of herbicide resistance mechanisms in plants. Target site resistance (TSR), where mutation/alteration in a gene encodes a structural change in its gene product so that herbicide cannot function in inhibitory manner, whereas, the phenomenon of non-target-site resistance (NTSR) is little understood and can impart unpredictable resistance by any mechanism not belonging to TSR (Délye, 2013; Sammons and Gaines, 2014). The use of glyphosate in agriculture has increased enormously since the introduction of glyphosate-resistant crop plants because it helps in weed control and thus improves the yield and profitability (Funke et al., 2006; Duke and Powles, 2008; Green, 2012). Further, glyphosate is a broad spectrum herbicide which inhibits the biosynthesis of the aromatic amino acids (tryptophan, tyrosine, and phenylalanine) by inhibiting the activity of EPSPS (5-enoylpruvyl shikimate-3-phosphate synthase), a key enzyme of shikimate pathway (Gomes et al., 2014). Literature reports that EPSPS overexpression provides high glyphosate tolerance in *Escherichia coli* and tobacco plants (Cao et al., 2013). Recently, Chhapekar et al. (2015) reported that overexpression of codon-optimized CP4-EPSPS helped rice to tolerate up to 1% commercial glyphosate. Further, stacking of Bt cry1Ah and mG2-epsps gene linked with LP4/2A showed higher expression and possessed good pest resistance and glyphosate tolerance in tobacco than those linked by 2A (Sun et al., 2012). The *EPSPS* gene has been isolated and characterized from mutant Rye grass (*Lolium rigidum*) (Simarmata and Penner, 2008) where mutation at nucleotide 301 (Cytosine to Thymine) of truncated *EPSPS* gene leads to change in amino acid code from Proline to Serine leading to the development of glyphosate tolerance. These observations confirm that *EPSPS* could be a good candidate for the development of herbicide tolerant crops. It will be beneficial to develop multi-stress tolerant transgenic plants which harbor tolerance to both salinity and herbicide.

Therefore, we designed a single construct containing both *PDH45* (salinity tolerance) + *EPSPS* (herbicide tolerance) genes and introduced it into tobacco plants. The transgenic plants overexpressed both the genes and provide salinity and herbicide tolerance. Furthermore, the literature suggests that transgenic plants overexpressing *PDH45* gene provide only salinity tolerance while *EPSPS* gene transgenics provide only herbicide tolerance.

## Materials and Methods

### Plasmids Construction and Transformation of Tobacco Plants

**FIGURE 1:**
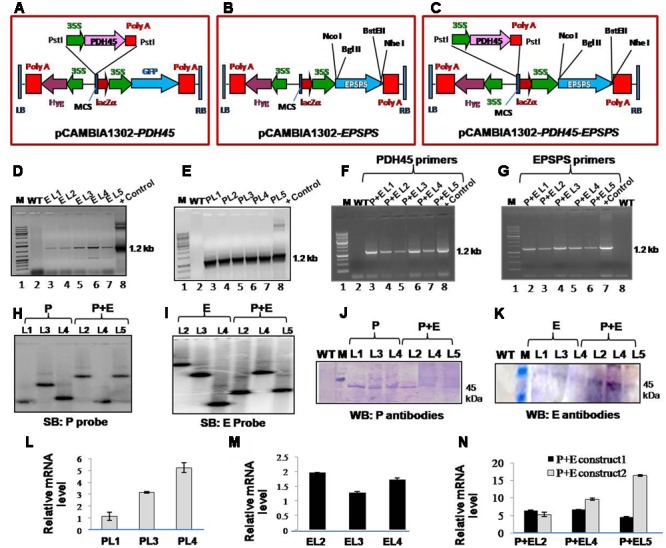
**Analysis of T_1_ transgenic lines (L1–L5) to confirm stable integration of transgene and the expression of gene. (A–C)** Schematic representation of map of pCAMBIA1302-*PDH45*, pCAMBIA1302-*EPSPS* and pCAMBIA1302-*PDH45+EPSPS* constructs used to overexpress *PDH45* and *EPSPS* cDNA in tobacco plants. **(D)** PCR confirmation of *EPSPS* T_1_ transgenic plants by using *EPSPS* (E) gene specific primers. **(E)** PCR confirmation of *PDH45* (P) transgenic plants by using *PDH45* gene specific primers. **(F)** PCR confirmation of PDH45*+EPSPS* (P+E) double transgenic plants by using *PDH45* gene specific primers. **(G)** PCR confirmation of *PDH45+EPSPS* double transgenic plants by using *EPSPS* gene specific primers. **(H,I)** Southern blot analysis to show the copy number of *PDH45* and *EPSPS* gene in tobacco transgenic plants. **(J)** Western blot analysis using anti-PDH45 antibodies. **(K)** Western blot analysis using anti-EPSPS antibodies. **(L)** qPCR analysis of *PDH45* transgenics with gene specific primers. **(M)** qPCR analysis of *EPSPS* transgenics with gene specific primers. **(N)** Expression analysis of *PDH45+EPSPS* overexpressing lines with *PDH45* and *EPSPS* genes specific primers. P+E construct 1 represents the expression analysis in *PDH45+EPSPS* transgenics with *PDH45* gene specific primers. P+E construct 2 represents the expression analysis in *PDH45+EPSPS* transgenics with *EPSPS* gene specific primers. P, E and P+E represent the same.

The complete ORF of *PDH45* cDNA (1.2 kb; Accession number Y17186.1) was PCR amplified using the gene specific primers (Supplementary Table S1) and cloned into pGEM-T easy vector (Promega). The *PDH45* gene fragment was isolated from pGEM-T-PDH45 plasmid and ligated into the MCS of pRT101 vector. The CaMV35S-PDH45-poly-A fragment from pRT101-PDH45 plasmid was isolated and ligated into the MCS of the binary vector pCAMBIA1302 which resulted in formation of construct pCAMBIA1302-PDH45 (**Figure 1A**). The Rye grass mutant *EPSPS* isolate from *Lolium rigidum* was PCR amplified (Supplementary Table S1) and cloned into pGEM-T easy vector. Further the *EPSPS* fragment was cloned into pCAMBIA1302 vector in place of GFP reporter, which resulted in the construction of a plasmid, pCAMBIA1302-EPSPS (**Figure 1B**). For the formation of dual gene (*PDH45*+*EPSPS*) construct, the 1.2 kb *EPSPS* gene fragment was cloned into pCAMBIA1302-PDH45 plasmid in place of GFP, which resulted in construction of a dual gene plasmid, pCAMBIA1302-PDH45-EPSPS (**Figure 1C**). Transformation of tobacco plants (*Nicotiana tabacum*) with *Agrobacterium tumefaciens* (LBA4404) containing the single or dual genes constructs (pCAMBIA1302-*PDH45*, pCAMBIA1302-*EPSPS*, or pCAMBIA1302-*PDH45-EPSPS*) was performed by using high frequency regeneration *via* direct somatic embryogenesis and efficient *Agrobacterium*-mediated genetic transformation method as described recently (Pathi et al., 2013). Competent strain of *Agrobacterium tumefaciens* (LBA4404) was transformed with the empty vector (pCAMBIA1302) construct as vector control (VC, the vector pCAMBIA-1302) using standard protocols. The empty vector contained all except the gene of interest. Non-transformed tobacco plant (*Nicotiana tabacum*) was also taken as control wild type (WT) plants.

### PCR and Southern Hybridization

Genomic DNA was isolated by CTAB method (*N*-acetyl-*N,N,N*-trimethyl ammonium bromide). PCR amplification was performed to amplify the 1.2 kb fragment of *PDH45* and *EPSPS* by using gene specific primers (Supplementary Table S1). Furthermore, 50 μg of genomic DNA from PCR positive transgenic lines was digested with XbaI for PDH45 and EPSPS, electrophoresed and blotted on nylon transmembrane (Amersham) as described (Sanan-Mishra et al., 2005). Complete radiolabeled ORF of *PDH45* and *EPSPS* cDNA was used as a probe.

### Western Blotting and qPCR Analysis

Total soluble proteins from the leaf tissues of WT and transgenic lines were resolved on to the 10% SDS-PAGE and further transferred to PVDF (Amersham) membrane followed by Western blot by using *PDH45* (Pham et al., 2000; Sanan-Mishra et al., 2005) and *EPSPS* (GenScript USA, Inc.) specific (1:10,000 dilution) antibodies.

The expression analysis of *PDH45* and *EPSPS* mRNA was analyzed by qPCR. Total RNA was isolated from 14 days old seedlings of WT and transgenic (*PDH45, EPSPS*, or *PDH45*+*EPSPS*) plants exposed to 200 mM NaCl by using TriZol reagent (Sanan-Mishra et al., 2005). Actin was used as an internal control and primers used for qPCR are listed in Supplementary Table S1. qPCR was done as described earlier (Garg et al., 2012).

### Leaf Disk Senescence Assay and Chlorophyll Estimation

Healthy and fully expanded leaves of WT, VC and transgenic tobacco plants (45 days old) were used to take the 10 mm circular disks. The disks were allowed to float in 6 ml NaCl and H_2_O (control) for 72 h and analyzed as described (Tuteja et al., 2013). Furthermore, the same disks were further used for chlorophyll estimation (Tuteja et al., 2013) and represented as % chlorophyll retained.

### Seedling Establishment and Growth Parameters

To check seedling establishment potential, root growth and fresh weight, the seeds of WT, VC and transgenic tobacco plants were chosen to determine the effects of salinity stress. Sterilized tobacco seeds of transgenic along with WT and VC were sown in Petri dishes (100 seeds per sample) containing full strength Murashige-Skoog (MS) solid medium (pH 5.8) supplemented with 200 mM NaCl and 30 mg/L hygromycin for salinity stress and selection marker, respectively. The number of germinated seeds was counted on daily basis, as it was defined as the emergence of the hypocotyls from 1 to 16 days. Measurement of root length (cm) and fresh weight (mg/plant) were taken at every sampling and all the measurements were carried out in triplicates. Seedlings stressed with 200 mM NaCl were later transferred to vermiculite pots and allowed to grow in green house (16 h light/8 h dark) at 25°C and were irrigated at every 10th day with 200 mM NaCl solution for 3 months.

### Glyphosate Tolerance by EPSPS Overexpressing Tobacco Plants

Seeds of various transgenics (*PDH45, EPSPS*, or *PDH45+EPSPS*), VC and WT were germinated on 1 mM glyphosate and fresh weights were recorded. The four to five leaf stage tobacco transgenic plants were sprayed with 1% (v/v) solution herbicide Roundup containing 41% glyphosate (Isopropyl amine salinity, Monsanto, Inc.) for different time points of post-treatment and accumulation of shikimic acid was estimated according to Cao et al. (2013).

### Measurement of ROS and Antioxidant Enzymes

Various oxidative stress markers [malondialdehyde (MDA); hydrogen peroxide (H_2_O_2_) and electrolytic leakage], and antioxidant enzymes [like catalase (CAT); ascorbate peroxidase (APX); glutathione reductase (GR)], proline and RWC (relative water content) were estimated in transgenic lines, VC and WT plants by using the methods described earlier (Garg et al., 2012). The nitroblue tetrazolium (NBT) (N6876, Sigma-Aldrich) and 3, 3 diaminobenzidine (DAB) (D5637, Sigma-Aldrich) stains were used to check the accumulation of superoxide radicals (O_2_^•–^) and H_2_O_2_, respectively (Dong et al., 2009). For O_2_^•–^ accumulation, seedlings were vacuum infiltrated with 0.1 mg ml^-1^ NBT in 25 mM Hepes buffer (pH 7.6) and incubated for 2 h at room temperature in the dark. The samples were then transferred to 80% ethanol and treated at 70°C for 10 min. For H_2_O_2_ accumulation, seedlings were vacuum infiltrated with 0.1 mg ml^-1^ DAB in 50 mM Tris-acetate buffer (pH 5.0) and incubated for 24 h at room temperature in the dark before transferring to 80% ethanol (Dong et al., 2009).

### Measurement of Photosynthetic Characteristics

Various photosynthetic parameters were recorded in fully expanded mature leaves using infra-red gas analyzer (IRGA, LiCor, Lincoln, NE, USA) during the sunny day between 10:00 and 11:00 h at atmospheric conditions.

### qPCR of SA and JA Pathway

To understand mechanism of stress tolerance in *PDH45* or *PDH45+EPSPS* overexpressing transgenic lines, the RNA from 3 to 5 days old WT, VC and transgenic tobacco seedlings was isolated. Sequence of 19 sets of genes related to different abiotic stress makers and hormonal pathways were downloaded from NCBI to design the primers (Supplementary Table S1) and qPCR was performed (Garg et al., 2012). For further confirmation of transgenics, VC and WT, seeds were grown in MS medium, 10 μM MeJ, 2 μM salicylic acid (SA), or combination of 10 μM MeJ+2 μM SA.

### Statistical Analysis

All the experimental data obtained are the means of three independent experiments under the same environmental condition and the results are shown as mean with standard error (mean ± SE). One way analysis of variance was used to test the significance between the mean values of control and transgenic plants and comparison among means was carried out using Tukey–Kramer multiple comparisons test with the help of Graph-Pad Instat software (ver 3.0). Statistically significant threshold was fixed to *P* < 0.05, *P* < 0.01, or *P* < 0.001.

## Results

### PDH45, EPSPS, or PDH45+EPSPS Overexpression in Tobacco Plants

To elucidate the functional significance of *PDH45* and *EPSPS* in one system, the complete ORF of pea *PDH45* (Sanan-Mishra et al., 2005) and Rye grass *EPSPS* were overexpressed in tobacco plants. As a VC, the only vector (pCAMBIA-1302) was transformed in tobacco and the transgenic plants were generated at the same time under the same conditions as the plants containing the vector along with *PDH45* and/or *EPSPS* the gene(s). The map of T-DNA single gene constructs (*PDH45* and *EPSPS*) and double gene construct (*PDH45*+*EPSPS*) used for raising transgenic tobacco plants were shown in (**Figures 1A–C**). The T_1_ and T_2_ generation plants segregated in the expected ratios [3:1 and 9:3:3:1, respectively (**Table 1**)]. Analysis of transgenic lines was performed in the T_1_ and T_2_ generations. A total of five hygromycin resistant transgenic plants with single gene overexpressing lines (*PDH45* or *EPSPS*) and double gene transgenics (*PDH45*+*EPSPS*) were confirmed by PCR using gene specific primers (Supplementary Table S1) and the expected size bands were obtained (**Figures 1D–G**). Three independent PCR positive lines of each set of transgenic plants were randomly selected for further analysis. Furthermore, the copy number of the integrated transgene in the single gene and double genes transgenic lines (T_1_) were subjected to Southern blot analysis. The single copy integration was observed in all the three lines tested for *PDH45* or *EPSPS* (**Figures 1H,I**). These results confirmed the integration of transgene (**Figures 1H,I**) in T_1_ tobacco plants. The results of western blot analysis clearly showed the presence of 45 kDa foreign protein in all the tested *PDH45* and *EPSPS* transgenic lines (**Figures 1J,K**), but no band was observed in WT plants with the same antibodies (**Figures 1J,K**). The specific transcript expression was observed in the entire single (*PDH45*, **Figure 1L**) (*EPSPS*, **Figure 1M**) and double transgenic plants (*PDH45+EPSPS*, **Figure 1N**); actin was used as internal control to normalize the expression intensity of all genes.

**Table 1:** Comparison of segregation ratio, seed number and weight of WT, VC, T_1_ and T_2_ transgenics of *PDH45, EPSPS*, and *PDH45+EPSPS* plants grown in the presence of H_2_O or 200 mM NaCl, respectively.

Parameter	Generation	H_2_O grown control plants		Transgenics grown in 200 mM salt		
		Wild type	Vector control	*PDH45*	*EPSPS*	*PDH45+EPSPS*
Segregation ratio, m^r^:m^s^ [n]	T_1_	0	3.2:1 (155)	3.18:1 (180)	3.22:1 (160)	3.11:1 (149)
Segregation ratio, m^r^:m^s^ [n]	T_2_	0	9.1:3.2:3.1:1(270)	9.1:2.18:3.1:1 (267)	9.31:2.33:3.38:1 (254)	9.22:3.76:3.12:1
Seed number per pod	T_1_	1567 ± 1.19^a^	1565 ± 1.15^a^	1568 ± 0.92^a^	1568 ± 1.76^a^	1566 ± 0.88^a^
Seed number per pod	T_2_	1160 ± 1.21^a^	1160 ± 1.54^a^	1163 ± 0.75^a^	1161 ± 0.99^a^	1164 ± 1.87^a^
Seed weight per pod (mg)	T_1_	117 ± 2.05^a^	118 ± 0.778^a^	117 ± 0.345^a^	115 ± 1.42^a^	118 ± 1.54^a^
Seed weight per pod	T_2_	124 ± 0.98^a^	123 ± 2.05^a^	124 ± 0.78^a^	125 ± 1.65^a^	127 ± 0.89^a^

*Each value represents mean of three replicates ± SE. Results are expressed as means with standard deviation (mean ± SD). The letters ‘a’ indicate non-significant differences at the level of *P* > 0.05, as determined by Tukey–Kramer multiple comparisons test with the help of Graph InStat software (version 3.0). m^*r*^: m^*s*^= Ratio between resistant and susceptible plants grown in hygromycin plate. [n] = Total number of plants mentioned for each line in brackets.*

### Performance of T_1_ Transgenic Plants under Salinity Stress

**FIGURE 2:**
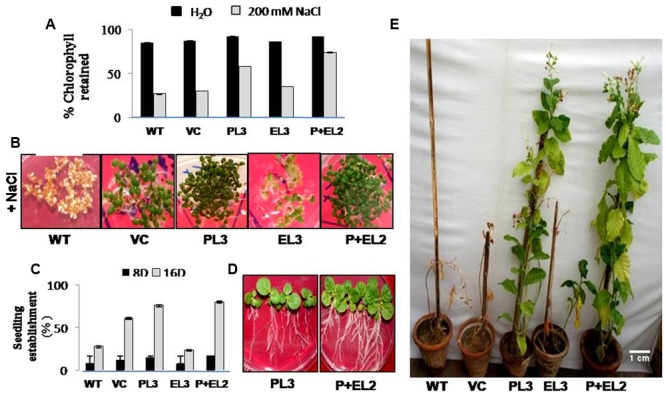
**Analysis of *PDH45* (P), *EPSPS* (E) and *PDH45+EPSPS* (P+E) dual gene construct tobacco transgenics in salinity stress (T_1_ generation). (A)** Quantification of retained chlorophyll in the leaf disk of transgenic lines, VC and WT used for leaf senescence assay under 200 mM salinity stress. **(B)** Seedling establishment of different transgenics (*PDH45, EPSPS* and *PDH45*+*EPSPS*) along with WT and VC in MS medium supplemented with 200 mM NaCl. **(C)** Representing % germination rate in transgenic lines at different time points 8 and 16 days of post-treatment of 200 mM NaCl. **(D)** Representative picture of transgenic tobacco (PL3 and P+EL2) seedling establishment. **(E)** Twenty days old tobacco transgenic seedlings (*PDH45, EPSPS*, and *PDH45*+*EPSPS*) were further transferred in vermiculite and irrigated with 200 mM NaCl at every 10 day for almost 3 months to further observe the effect of salinity stress on transgenics as compared to WT plants.

To validate the salinity tolerance of single gene and double gene transgenics, leaf disk from the T_1_ transgenics and WT plants were floated separately in 200 mM NaCl and H_2_O (as control) for 72 h. Salinity stress accompanied lesser loss of chlorophyll content in *PDH45* or *PDH45+EPSPS* transgenic plants as compared to WT, VC and *EPSPS* alone plants (**Figure 2A**). These results explored the better ability of *PDH45* or *PDH45+EPSPS* transgenic plants in confronting salinity stress. Phenotypic comparison with 200 mM NaCl stress showed that WT, VC and EPSPS overexpressing seed did not germinate efficiently, whereas, *PDH45* or *PDH45+EPSPS* overexpressing seeds germinated efficiently (**Figure 2B**). **Figures 2A,B** represent the results of one line of each transgenic plant; the other two lines also showed the same result. The *PDH45* or *PDH45+EPSPS* transgenic lines also showed higher germination potential at 8th and 16th days as compared to WT, EV and *EPSPS* alone plants under NaCl stress (**Figure 2C**). Interestingly, under 200 mM NaCl stress, the growth of the *PDH45+EPSPS* transgenic seedlings was found to be better as compared to *PDH45* alone transgenic seedlings (**Figure 2D**). Beyond that, salinity stress also resulted in reduced fresh weight and root length of WT, VC and *EPSPS* transgenic plants as compared to *PDH45* or *PDH45+EPSPS* transgenics (**Table 2**). Further to study the effect of *PDH45* or *PDH45+EPSPS* overexpression in transgenic tobacco plants under salinity stress, the seedlings were transferred to soil and irrigated with 200 mM NaCl (every 10th day) for 3 months and several growth parameters such as plant height, fresh weight of leaves and time required for flowering were observed to be higher in *PDH45* or *PDH45+EPSPS* (**Table 2**) tobacco transgenic plants in comparison to WT, VC and *EPSPS* only transgenic plants. Interestingly, it was observed that WT, VC or *EPSPS* transgenic plants failed to sustain growth in the presence of NaCl and eventually died, while the *PDH45* or *PDH45+EPSPS* transgenic plants maintained regular growth rate, reached to maturity on time, flowered and set viable seeds (**Figure 2E** and **Table 1**). Overall, the *PDH45* or *PDH45+EPSPS* lines grew normally under continuous stress and produced a yield comparable to WT plants grown in H_2_O. However, WT plants did not survive until maturity under continuous stresses. These findings indicate that *PDH45* or *PDH45*+*EPSPS* overexpression promotes salinity tolerance in tobacco.

**Table 2:** Comparison of various yield parameters obtained in transgenic (T_1_ and T_2_ generation), wild type and vector control plants grown under H_2_O and 200 mM NaCl, respectively.

Parameter	Generation	H_2_O grown control plants		Transgenics grown in 200 mM NaCl		
		Wild type	Vector control	*PDH45*	*EPSPS*	*PDH45+EPSPS*
Fresh weight of leaves	T_1_	0.57 ± 1.23^b^	0.40 ± 0.67^c^	4.50 ± 2.13^a^	0.45 ± 0.95^c^	4.8 ± 0.36^a^
Fresh weight of leaves	T_2_	0.50 ± 1.56^d^	0.50 ± 1.65^d^	2.34 ± 0.087^a^	0.60 ± 0.87^c^	1.9 ± 0.55^b^
Root length (cm)	T_2_	0.25 ± 0.61^d^	0.30 ± 1.41^c^	0.80 ± 1.08^b^	0.30 ± 0.56^c^	1.2 ± 1.01^a^
Plant height (cm)	T_1_	35.6 ± 1.9^c^	42.0 ± 0.99^bc^	85 ± 1.02^a^	46.6 ± 0.32^b^	87 ± 2.13^a^
Plant height (cm)	T_2_	48.0 ± 2.34^c^	43.0 ± 1.54^d^	87 ± 0.78^a^	54 ± 1.54^b^	87.4 ± 3.01^a^
Flowering time (D)	T_1_	119 ± 1.12^b^	130 ± 1.04^a^	112 ± 1.06^c^	117 ± 1.15^c^	109 ± 2.11^c^
Flowering time (D)	T_2_	125 ± 1.23^a^	128 ± 1.65^a^	120 ± 1.68^a^	128 ± 1.15^a^	115 ± 1.09^b^

*Each value represents mean of three replicates ± SE. Results are expressed as means with standard deviation (mean ± SD). One way analysis of variance was used to test significance between mean values of WT and transgenic plants. Comparison among means was carried out using Tukey–Kramer multiple comparisons test with the help of Graph InStat software (version 3.0). The transgenic lines at *P* < 0.05, *P* < 0.01, and *P* < 0.001 were considered as statistically significant. The letters a, b, c, and d indicate significant differences.*

### EPSPS or EPSPS+PDH45 Overexpressing Transgenic Tobacco Plants Showed Glyphosate Tolerance

**FIGURE 3:**
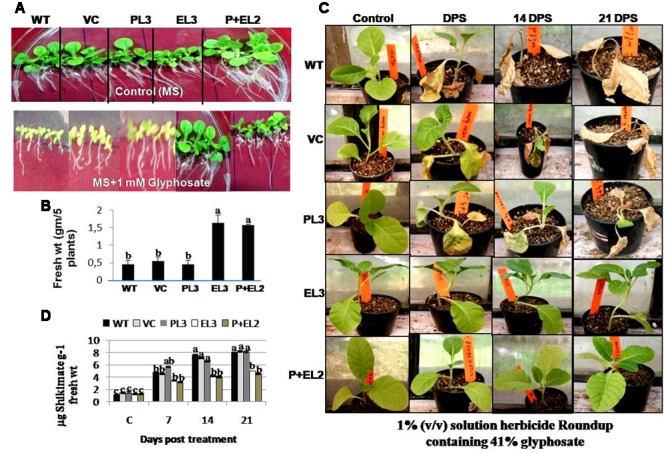
**Glyphosate tolerance of *PDH45* (P), *EPSPS* (E) and *PDH45+EPSPS* (P+E) transgenics, VC and WT plants in green house, when sprayed with 1% (v/v) solution herbicide Roundup containing 41% glyphosate. (A)** T_1_ tobacco seedlings were germinated on the plain MS and MS containing 1 mM glyphosate and grown for 7 days at 100 μmol m^-2^ with 16 h L/8 h dark period. **(B)** Fresh weight of seedlings was recorded. **(C)** These seedlings were further transferred to the soil in pots and grown for another month. Six to eight leaf stage transgenic plants were sprayed with 1% (v/v) solution herbicide Roundup containing 41% glyphosate. After 7 DPS (days post spray), 14 and 21 DPS, injury was observed. Photographs of tobacco seedlings have been taken after spray at above mentioned time points. Plants used for spray were further used for shikimate estimation. **(D)** Accumulation of shikimate content was determined in transgenic lines and WT plants. Experiment was repeated twice in three independent plants. Data represent the means ± SD of three independent experiments (*n* = 3), ^a^*P* < 0.05, ^b^*P* < 0.01, ^c^*P* < 0.001.

All the transgenics, WT or VC tobacco seedlings were grown vertically on the MS agar medium with or without 1 mM glyphosate for 10–12 days. All the transgenic plants, WT or VC showed good growth on MS agar medium, however, the double genes transgenic (*PDH45+EPSPS*) performed superior (**Figure 3A**). But treatment with 1 mM glyphosate inhibited the growth of WT, VC or transgenic plants overexpressing *PDH45* (**Figure 3A**). Plants overexpressing *EPSPS* or *PDH45+EPSPS* genes grew more quickly and remained green till 10th day after treatment with 1 mM glyphosate. The fresh weight of five independent transgenic plants for each gene was measured to assess the inhibition of plant growth by glyphosate (**Figure 3B**). The transgenic plants overexpressing *EPSPS* or *PDH45+EPSPS* genes had the highest fresh weight as compared to WT, VC and *PDH45* transgenics (**Figure 3B**). To further confirm the glyphosate tolerance amongst the different transgenic plants, 6–8 leaf stage tobacco transgenic along with WT or VC plants were sprayed with 1% (v/v) solution herbicide Roundup containing 41% glyphosate at equal dose. The injury caused by glyphosate was recorded after 7, 14, or 21 days of post spray (DPS). All WT, VC or *PDH45* (**Figure 3C**) transgenic plants showed severe injury and eventually died, whereas, transgenic plants containing *EPSPS* or *PDH45+EPSPS* genes remained healthy (**Figure 3C**). Furthermore, the leaves of same plants were used for shikimate estimation at 7, 14, or 21 DPS. In the present study, EPSPS alone or EPSPS + PDH45 overexpressing transgenic lines showed less affinity to glyphosate treatment may be because of truncated EPSPS gene. Therefore, glyphosate treatment does not have any effect on shikimate level and remained comparable to the control treatment without glyphosate. The results show that the truncated EPSPS gene is lesser impacted by glyphosate. Before treatment, WT, VC or all the transgenic plants showed similar shikimate levels, while treatment with 1 mM glyphosate increased the levels of shikimate in leaves of WT, VC or *PDH45* transgenic plants up to 21 DPS (**Figure 3D**). However, significantly lesser accumulation of shikimate was observed in *EPSPS* or *PDH45*+*EPSPS* transgenics (**Figure 3D**). These results clearly confirm major role of the *EPSPS* gene in providing herbicide tolerance.

### Dual Gene (PDH45 + EPSPS) Transgenic Plants Accumulate Less MDA, H_2_O_2_ and Ion Leakage and Show Better Antioxidant and Photosynthetic Response under Salinity Stress

**FIGURE 4:**
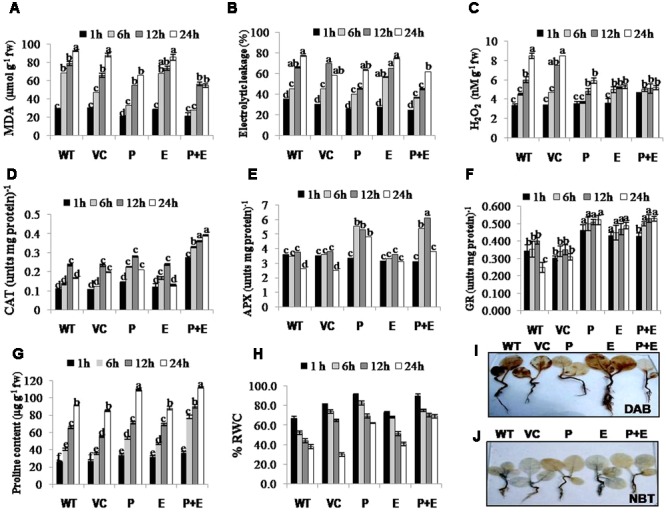
**Biochemical analysis of *PDH45* (P), *EPSPS* (E) and *PDH45+EPSPS* (P+E) transgenics, VC and WT plants under 200 mM NaCl conditions. (A)** Determination of lipid peroxidation expressed in terms of MDA content in transgenic lines, WT and VC. **(B)** Estimation of percentage electrolytic leakage in transgenic lines, WT and VC. **(C)** Changes in the level of hydrogen peroxide (H_2_O_2_) content in transgenic lines along with WT and VC. **(D)** Catalase (CAT) activity in transgenic lines, WT and VC. One unit of enzyme activity is defined as 1 μmol H_2_O_2_ oxidized min^-1^. **(E)** Changes in ascorbate peroxidase (APX) enzyme activity in transgenic lines, WT and VC. One unit of enzyme activity defined as 1 μmol of ascorbate oxidized min^-1^. **(F)** Changes in glutathione reductase (GR) enzyme activity in transgenic lines, WT and VC. One unit of enzyme activity is defined as 1 μmol of GS-TNB formed min^-1^ due to reduction of DTNB. **(G)** Changes in the level of proline accumulation in transgenic lines, WT and VC. **(H)** Estimation of percent relative water content (RWC) in *PDH45, EPSPS* and *PDH45+EPSPS* transgenic lines, WT and VC. **(I)** Detection of hydrogen peroxide (H_2_O_2_) accumulation using 3, 3-diaminobenzidine (DAB) staining method. **(J)** Detection of superoxide radical (O_2_^•–^) accumulation using nitroblue tetrazolium (NBT) staining method. Data represent the means ± SD of three independent experiments (*n* = 3), ^a^*P* < 0.05, ^b^*P* < 0.01, ^c^*P* < 0.001.

Salinity induced changes in the accumulation of MDA (malondialdehyde; by product of lipid peroxidation), H_2_O_2_ and ion leakage were quantified in T_1_ tobacco transgenics along with WT or VC plants as important stress biomarkers. Significantly reduced levels of MDA, ion leakage or H_2_O_2_ (**Figures 4A–C**) accompanied with induced activities of antioxidant enzymes such as CAT, APX, or GR (**Figures 4D–F**) were observed in *PDH45* or *PDH45+EPSPS* transgenic plants as compared to WT, VC or *EPSPS* transgenic plants. As a part of antioxidant defense in plants, proline accumulation was also found to be higher in *PDH45* or *PDH45+EPSPS* transgenic lines as compared to WT, VC or *EPSPS* transgenic plants (**Figure 4G**), which further led to efficient maintenance of higher water balance (**Figure 4H**) under salinity stress.

**FIGURE 5:**
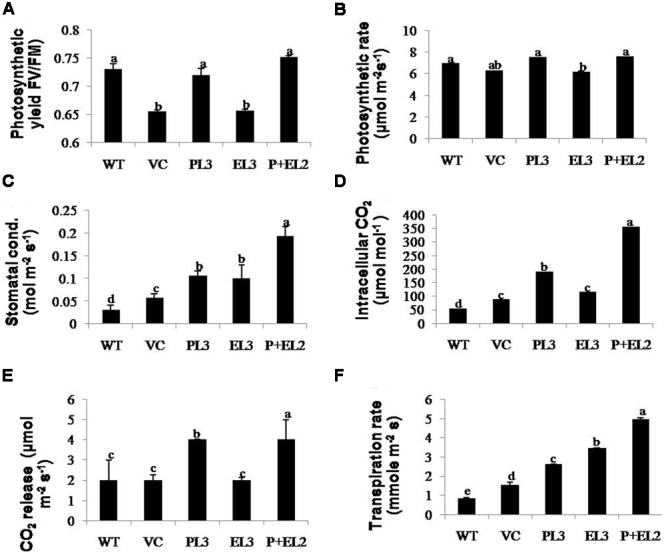
**Determinations of changes in photosynthetic machinery were performed by IRGA in transgenics including *PDH45* (P), *EPSPS* (E) and *PDH45+EPSPS* (P+E), VC and WT plants under salinity stress.** Fully expanded leaves were chosen for measurement of photosynthetic activity under constant sunlight. **(A)** Photosynthetic yield FV/Fm. **(B)** Measurement of photosynthesis rate. **(C)** Stomatal conductance. **(D,E)** Quantification of intracellular and released CO_2._ **(F)** Measurement of transpiration rate against 200 mM salinity stress. Data are shown as the average of ±SD of three independent transgenic plants, *P* < 0.05, *P* < 0.01, *P* < 0.001. The letters a, b, c, d, and e indicate significant differences.

H_2_O_2_ detection and quantification were performed using 3, 3-diaminobenzidine (DAB) staining method. Polymerization of DAB, visible as brown precipitate against H_2_O_2_ was detected in WT, VC or all the transgenic tobacco plants, while significantly lesser H_2_O_2_ accumulation was detected in *PDH45* or *PDH45+EPSPS* plants as compared to WT, VC or *EPSPS* overexpressing lines (**Figure 4I**), which showed marked increase in H_2_O_2_ accumulation under salinity treatment (**Figure 4I**). Besides that O_2_^•–^ detection and quantification were performed using NBT staining method after 24 h of salinity treatment. The results revealed accumulation of O_2_^•–^ in the form of blue color in all transgenics or WT plants; the blue color was comparatively more in *EPSPS* transgenic plants along with WT and VC (**Figure 4J**). Furthermore, the accumulation pattern of O_2_^•–^ and H_2_O_2_ shown by NBT and DAB staining can be arbitrary and only reflective in nature. The NBT and DAB tests were performed after salinity stress in control and transgenic lines and may not reflect as under natural conditions. Parameters of photosynthesis efficiency were quantified after exposure to 24 h salinity stress in tobacco transgenics along with WT and VC plants. Net photosynthetic rate, yield, stomatal conductance or transpiration rates were found to be higher in *PDH45* and *PDH45+EPSPS* transgenic plants in comparison to WT, VC or *EPSPS* transgenic plants (**Figures 5A–C,F**). Moreover, *PDH45* or *PDH45+EPSPS* transgenic plants showed marked increase in respiration (**Figures 5D,E**), indicating higher and constant photosynthetic rates even under salinity stress, whereas, WT, VC or *EPSPS* transgenics plants were strongly affected.

### PDH45 Modulates Jasmonic and Salicylic Acid Signaling Pathways for Salinity Tolerance

**FIGURE 6:**
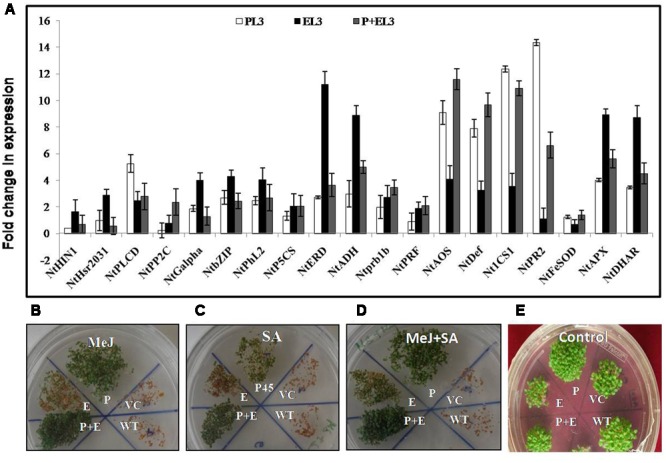
**(A)** qPCR analysis of various abiotic stress marker and hormonal pathway related genes (*NtHin1, NtHsr2031*: cell death marker *NtPLCD*; *NtPP2C*; *NtGalpha*: upstream of ABA signaling, *NtbZIP*; *NtPhl2*: transcription factors in ABA signaling, *NtP5CS*; *NtERD*; *NtADH*: ABA responsive genes, *NtADH*; *NtPrb1b*; *NtPRF*: ethylene pathway marker genes, *NtAOS*; *NtDef*: jasmonic acid pathway marker genes, *NtICS1*; *Nt PR2*: salicylic acid pathway marker genes, *NtFeSOD*; *NtAPX*; *NtDHAR*: ROS pathway marker genes) in *PDH45* (P), *EPSPS* (E), and double construct containing *PDH45+EPSPS* (P+E). **(B–E)** Seedling establishment of all transgenic lines along with WT and VC plants in 10 μM MeJ, 0.2 mM SA, and 10 μM Mej+0.2 mM SA hormones. **(B)** Seedling establishment in MeJ. **(C)** Seedling establishment of transgenic lines along with VC in SA. **(D)** Seedling establishment of transgenic lines along with VC in Mej+SA. **(E)** Seedling establishment of transgenic lines along with VC in Plain MS medium as a control.

Finally, to study the effect of *PDH45* overexpression in transgenic lines on the expression of stress and hormonal markers, we designed 19 pair of primers (Supplementary Table S1) related to different abiotic stress marker and hormonal pathways related genes to study their expression level in transgenic plant (*PDH45, EPSPS*, or *PDH45+EPSPS*). Genes related to cell death marker, upstream of ABA signaling, transcription factors in ABA signaling, ABA responsive genes, ethylene pathway marker genes, jasmonic acid (JA) pathway, SA and ROS pathway marker genes were found to be significantly induced in all the transgenic (**Figure 6A**) under salinity treatment. Interestingly, especially for salinity treated *PDH45* or *PDH45+EPSPS* transgenic plants, expression level of JA pathway related genes including *NtDEF* and *NtAOS* (allene oxide synthase) were significantly higher than the expression of other pathway related genes. Moreover, SA pathway genes such as *NtICS1* and *NtPR2* also responded effectively with genes related to JA pathway in operating salinity tolerance mechanism in *PDH45* or *PDH45+EPSPS* transgenic plants. However, *EPSPS* overexpressing plants were observed to utilize ABA (*NtADH* and *NtERD*) and ROS pathway (*NtAPX* and *NtDHAR*) marker genes to defend against stress. For further confirmation of MeJ and SA mediated signaling pathway opted by *PDH45* or *PDH45+EPSPS* against salinity stress, we germinated the seeds of all transgenic lines along with WT and VC plants in MS medium (control) and MS medium supplemented with MeJ, SA, or MeJ+SA. In the presence of MeJ, SA or MeJ+SA hormones, the *PDH45, PDH45+EPSPS* showed early and efficient germination rate as compared to WT, VC or *EPSPS* transgenic plants (**Figures 6B–D**). However, in control conditions all the transgenics, VC or WT had similar germination rate (**Figure 6E**).

### Analysis of T_2_ Generation Transgenic Plants

The T_2_ generation transgenic plants from all the lines (*PDH45, EPSPS*, or *PDH45+EPSPS*) were also raised and found positive as checked by gene specific primers (Supplementary Figures S1A–D). The T_2_ transgenic seeds of *PDH45* or *PDH45+EPSPS* transgenic plants germinated and grew efficiently under 200 mM NaCl, while the WT, VC or *EPSPS* transgenic seeds germinated weakly and showed reduced growth and turned yellow (Supplementary Figure S1E). In the leaf disk assay, the salinity stress induced loss of chlorophyll was quantified in T_2_ transgenics. As a result, % retained chlorophyll was higher in *PDH45* or *PDH45+EPSPS* transgenic plants in comparison to WT, VC or *EPSPS* overexpressing lines, under 200 mM NaCl as checked by quantification of chlorophyll content in the leaf disks after 72 h (Supplementary Figure S1F).

## Discussion

Being sessile, plants are continuously encounter abiotic stress conditions that impose serious detrimental effects and cause significant decline in crop productivity (Gururani et al., 2015). Among abiotic stresses, high salinity, weed stress or herbicide stress cause serious alteration in growth responses and damage to photosynthetic machinery. Plants have evolved several mechanisms to perceive and transmit signals to cellular machinery that eventually activates responses in terms of abiotic and biotic stress management (Thomashow, 1999; Xiong et al., 2002; Mahajan and Tuteja, 2005). The understanding of stress tolerance mechanism against various stress factors may prove beneficial for developing tolerant crop plants. Hence, in this context, we have earlier shown that overexpression of single gene (*PDH45, PDH47, SUV3*, or *p68*) in tobacco and rice transgenic plants resulted in high salinity stress tolerance without any yield penalty (Sanan-Mishra et al., 2005; Vashisht et al., 2005; Amin et al., 2012; Gill et al., 2013; Tuteja et al., 2013, 2014; Banu et al., 2015). In addition to the salinity stress, weeds are also considered as one of the major problem for agricultural productivity. Regarding that *EPSPS* has been reported as a promising gene for its role in glyphosate effect (Cao et al., 2013; Délye et al., 2013). Therefore, these findings encouraged us to generate transgenic plants having dual characteristics of salinity and herbicide tolerance, harboring both the *PDH45* and *EPSPS* genes as salinity and herbicide tolerant genes, respectively. In the era of frequently changing environmental conditions, there is an urgent need of developing crops with multiple stress tolerance features. Therefore, our research provides novel insights into developing transgenics with multiple traits of salinity tolerance and herbicide resistance without the yield loss.

The *PDH45* alone and *PDH45+EPSPS* double construct overexpressing transgenic tobacco plants showed tolerance against salinity as observed by higher chlorophyll retention and percent germination potential, in comparison to WT, VC and *EPSPS* overexpressing plants. Moreover, T_1_ seedlings of *PDH45* and *PDH45+EPSPS* transgenics plants were able to survive, flower and set viable seeds even under salinity stress. These observations suggest that foreign trait is functionally active and stable in transgenic plants. In addition to salinity stress treatment, glyphosate spray on different transgenic and WT, VC plants revealed that the *EPSPS* overexpressing transgenic plants showed lesser injury and survived better, whereas, *PDH45*, WT and VC were injured, dried and finally could not survived. The treatment of plants with glyphosate might have inhibited the *EPSPS* activity and led to the accumulation of shikimate (Barry and Padgette, 1992; Chang et al., 2003; Délye et al., 2013). Moreover, it has been observed that glyphosate treatment caused accumulation of shikimate in glyphosate insensitive tobacco cells, whereas, lack of shikimate accumulation was found in tolerant cells (Tian et al., 2010). In tune with above studies, treatment with 1 mM glyphosate induced the accumulation of shikimate in both WT and transgenic plants but the accumulation of shikimate was lower in transgenic plants overexpressing *EPSPS* gene. This finding confirmed the role of *EPSPS* gene as an active target for glyphosate action. Previously reported studies also focused on isolation and characterization of *R. aqualilis* strain *EPSPS* in tobacco significantly reduced the glyphosate sensitivity (Peng et al., 2012). Besides that, Cao et al. (2013) proved the role of five bacterial gene coding for *EPSPS* in the *E. coli* and transgenic tobacco against glyphosate. Overexpression of codon-optimized CP4-EPSPS in rice helped the transgenic plants to tolerate up to 1% commercial glyphosate treatment (Chhapekar et al., 2015). Further, stacking of Bt cry1Ah and mG2-epsps gene linked with LP4/2A showed higher expression and possessed good pest resistance and glyphosate tolerance in tobacco than those linked by 2A (Sun et al., 2012).

It has been proposed that ROS accumulation (O_2_^•–^, H_2_O_2_, etc.) during salinity stress caused accumulation of MDA, damage to cellular macromolecules and eventually affects the stability of membranes (Apel and Hirt, 2004; Gill and Tuteja, 2010; Gill et al., 2012; Das and Roychoudhury, 2014). In the present study, we showed that *PDH45* and *PDH45+EPSPS* transgenic plants represent less O_2_^•–^ and H_2_O_2_ accumulation thus significantly lesser lipid peroxidation, H_2_O_2_ content and ion leakage along with increased activities of antioxidant enzymes including CAT, APX, and GR. The efficient ROS scavenging activities of antioxidant enzymes triggered by ROS and higher accumulation of osmoprotectants in *PDH45* and *PDH45+EPSPS* transgenic plants could be one of the reasons in maintaining the integrity of membranes for the plant survival under the stress. Our results and previously reported studies in rice (Gill et al., 2013) also suggest delayed senescence in *PDH45* overexpressing transgenic plants which was co-related with increased tolerance to oxidative stress.

*PDH45* and *PDH45+EPSPS* overexpressing transgenic plants showed lesser damage to photosynthetic apparatus, thus maintaining the normal growth and yield of transgenic plants, whereas, WT and VC experienced maximum damage to photosynthesis machinery under 200 mM NaCl stress. In general the reduction in photosynthesis with increased salinity stress could be attributed to the difference in the efficiency of root system in limiting the transport of ions to shoots (Mahajan and Tuteja, 2005; Munns et al., 2006; Gill et al., 2013). Furthermore, the inefficiency of photosynthesis under salinity stress may be due to stomatal exposure under water deficiency (Meloni et al., 2003) in addition to impaired several biochemical and physiological process like imbalance between ROS and their scavenging system (Sultana et al., 1999; Gill and Tuteja, 2010; Das and Roychoudhury, 2014).

In order to gain insights regarding pathway utilized by *PDH45* and *PDH45+EPSPS* transgenic plants in tolerating salinity stress, we performed expression studies for 19 genes related to abiotic stress marker and hormonal pathways. However, the pathways, utilized by *PDH45* gene in providing salinity tolerance is still obscure. Our studies showed the maximum transcript accumulation AOS (allene oxide synthase) and *NtDef* genes related to JA pathways and demonstrate that *PDH45* and *PDH45+EPSPS* transgenic plants can induce JA production and could promote the accumulation of secondary metabolites for the survival of plants under salinity stress. As an important signal molecule, JA (endogenous regulator) plays a key role in regulating stress responses, plant growth and development (Turner et al., 2002; Qiu et al., 2014). It has been reported that the exogenous application of 2 mM JA on wheat seedlings under salinity stress resulted in significant decrease in MDA, H_2_O_2_ content, O_2_^•–^ radical production and significant increase in the transcript levels of SOD, POD, CAT and APX and GSH content, chlorophyll b and carotenoids content (Qiu et al., 2014). In addition to JA, SA also acted as a signal molecule in providing salinity tolerance to *PDH45* and *PDH45+EPSPS* transgenic plants. Cross-talk between different signal transduction pathways as opposed to single signaling pathways, mediates gene expression and production of secondary metabolites during plant defense responses (Reymond and Farmer, 1998; Mur et al., 2006; Khan et al., 2015).

Interestingly, present work reports the synergistic effect of JA related genes with SA signaling pathways genes. Though, JA is commonly postulated to act antagonistically on the SA regulating pathways and on the expression of SA dependent genes (Kachroo et al., 2001; Kloek et al., 2001). In parallel to that, both JA and SA are also considered as the important signal molecules in plant defense responses (Kunkel and Brooks, 2002; Shah, 2003; Qiu et al., 2014; Khan et al., 2015). Overall results suggest that *PDH45* and *PDH45+EPSPS* may play a critical role in plant response against salinity stress and might act as a component of JA and SA mediated signaling pathways. However, the confirmation of JA and SA pathways modulated by *PDH45* and *PDH45+EPSPS* in tobacco response to salinity stress need to be investigated further. Thus, finally it can be concluded that pyramiding of the *PDH45* gene with *EPSPS* gene renders host plants tolerant to salinity and herbicide by enhancing antioxidant machinery through modulation of JA and SA mediated signaling pathways.

## Gene Bank Accession Numbers

*PDH45* – Y17186.1, *EPSPS* – US2007/0180574A1.

## Author Contributions

The experimental set up was designed by NT, RT, BG, NK and SG and BG, DB, RS performed the experiments and acquired the data. NT, RT, BG, NK, and SG analyzed the data. BG, DB, NT, RT, NK, and SG interpreted the results and wrote the manuscript. NT and RT supervised the project.

## Conflict of Interest Statement

The authors declare that the research was conducted in the absence of any commercial or financial relationships that could be construed as a potential conflict of interest.
