# Supplementary material for: Simultaneous Expression of PDH45 with EPSPS Gene Improves Salinity and Herbicide Tolerance in Transgenic Tobacco Plants
Source: Front Plant Sci. 2017 Mar 24;8:364. doi: 10.3389/fpls.2017.00364 (PMC5364135; doi:10.3389/fpls.2017.00364)
Supplement: Supplementary file 1 [file Data_Sheet_1.doc]

**Supplementary Table S1**. Primers used for PCR and qRT-PCR analysis.

| **S. No.** | **Primer ID** | **Gene Bank Acc. No/US Patent** | **Sequence of the primers: Forward (5’-3’)/ Reverse (5’-3’)** |
| --- | --- | --- | --- |
| 1 | *PDH45* | Y17186 | ATGGCGACAACTTCTGTGGTGC TTATATAAGATCACGAATATTCATTGGCATCTG |
| 2 | *EPSPS* | US2007/0180574A1 | FP1- GATGCCAAGGAGGAAGTAAAG, FP2 – GCTATCAGATGTTGCGTCCTG, RP1 – CTCTGGTGGCGTGATAATG, RP2 – AACAGGTGGGCAGTCAGTG, RP3 – ATAGGACGCTCCCTCATTCTTGGT, RP4 - TTTCCACCAGCAGCTACAGCA, dT-Anchor – GACCACGCGTATCGATGTCGACTTTTTTTTTTT, Anchor Primer - GACCACGCGTATCGATGTCGAC |
| 3 | *PDH45* (for RT) | Y17186 | GCCGAGGTACTAAGGAGTCAATAATT**/**  GAAAGCATGGTTATAGAAT |
| 4 | *EPSPS* (for RT) | US2007/0180574A1 | CCGCAGCAGCGTTATCTACA**/**  GAGGTGCTTCTCTGCAGCCG |
| 5 | *NtHin1* | AF212183 | CGTCCTAACAAAGTCAAGTTCTACGTGAC**/**  CCTCTCGCCTTGATACAAAGCTCTAGC |
| 6 | *NtHsr2031* | AF212184 | GCTGATTGGTTCATGTACTACACTGTCTACACG**/**  CAGAGGAGA GCGGCGAAA CC |
| 7 | *NtPLCD* | Z84822 | GACGGTTTGATGGCCACTCATGA**/**  TCCACTACCACGATTTTCTGGTGATGC |
| 8 | *NtPP2C* | AJ309007 | GGAAGACGACGTGATATGGAAGATGC**/**  TGCATTCTATCTTTGCATTTCATAGCCACATG |
| 9 | *NtbZIP* | DQ073639 | CACTACCTCGGACGCTTAGTCAGAAAAC**/**  GGTTGCATATCTTCTCGAACCACTCCTG |
| 10 | *Ntphi-2* | AB063648 | CGTTGGCTCGACAATCTTCCATATACTCG**/** CTGGCACACTTACATTTCCACCAGC |
| 11 | *NtP5CS* | HM854026 | GAGTTGGTCGTCAGCGGCTTAGATA/ GCTGAGCTGATGTCATATCCAACTGACTG |
| 12 | *NtERD10B* | AB049336 | CTCTAGCTCTTCGGAGGATGATGGA/ CCTTCCATACCATAACCGGTAGTCGTAG |
| 13 | *NtADH* | AY619947 | CTCTCTGCCATACTGATGTCTACTTCTGG**/**  CTTTCTTCCGATTTGCAGTGAGCACAATC |
| 14 | *Ntprb-1b* | X66942 | CCGATAAAGTCTGCTAACATGTGACTTTCCAA**/**  AAC TTG TCT ACG AGC TGC ATT GTG AGG |
| 15 | *NtPRF* | AF154685 | GATGATGTTGACAGCAGAGAAAGTGTGC**/** CAACCAATTCTCCTTGGCTGCGATC |
| 16 | *NtAOS* | AB233414 | CCGATCCTTTTCGACGTTTCTAAAGTCG**/**  ACA GCT CGG TAT AGC TTT CAT GGA ATT G |
| 17 | *NtDEF* | X99403 | GCGCTTCTTTGCAACTGTGTTACTTATAGC**/** TCAGACAAACGGTGGCACAGTTG |
| 18 | *NtICS1* | AY740529 | GAGTTGTGACAGCTGCGGACATTG**/**  TGT TCC AGC TAA AGC CTC GCT ACA |
| 19 | *NtPR2* | M60460 | CCACTTCAAGATCTTCAATCCCTAAC/ GGCAGGAGCAACAAATGGTGC |
| 20 | *NtFeSOD* | M55909 | GAGCTGCTTAGCGAGAAGACATGGA**/** CCTCCAAATACAGAAAATCACTTGACCCTCC |
| 21 | *NtAPX* | U15938 | GGTGATTTCTATCAATTAGCTGGAGTTGTTGC**/**  CATTTGCTTCACAAACACATCTCTCAAGTGG |
| 22 | *NtDHAR* | AY074787 | CTTGGAAGTGAACCCAGAAGGAAAAGTTC**/**  ACTAGCATCCTTGCTCTTCCGAAATGAGA |

***PDH45***-Pea DNA helicases, ***EPSPS***-5-enoyl pyruvyl shikimate phosphate synthase, ***NtHin1****-*Hairpin inducing protein, ***NtHsr2031****-* Cell death inducing protein, ***NtPLCD****-* Phospholipase D, ***NtPP2C****-* Protein phosphatase 2C, ***NtbZIP****-* Basic leucine zipper protein, ***Ntphi-2****-* Phosphate induced like gene, ***NtP5CS****-* 1- Pyrroline-5-carboxylate synthetase, ***NtERD10B****-* Dehydrin like protein, ***NtADH****-* ADH like UDP-glucose- dehydrogenase mRNA, ***Ntprb-1b****-* Basic pathogen related, ***NtPRF****-* 12S ribosomal RNA, mitochondrial gene, ***NtAOS****-* Allene oxide synthase, ***NtDEF****-* Defensin related gene, ***NtICS1****-* Isochorismate synthase protein, ***NtPR2****-* Pathogen related gene, ***NtFeSOD****-* iron superoxide dismutase, ***NtAPX****-* Ascorbate peroxidase, ***NtDHAR****-* Dehydroascorbate peroxidase, **RT**- real time PCR.

**Supplementary Figure S1**


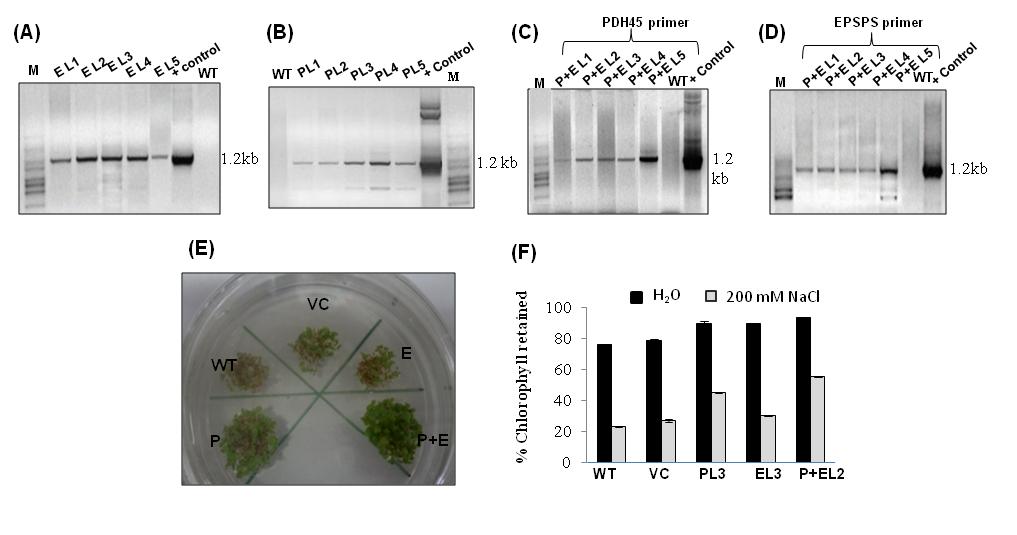


**Supplementary Fig. S1.** Analysis of T_2_ transgenic plants upon exposure to 200 mM NaCl. **(A)** PCR amplification for overexpressing *EPSPS* E) plants by using *EPSPS* gene specific primers. **(B)** PCR confirmation of over expressing *PDH45* (P) plants by using gene specific reverse primer. **(C and D)** Showing PCR confirmation for *PDH45+EPSPS* (P+E) double constructs transgenics by using *PDH45* and *EPSPS* gene specific primers, respectively. **(E)** Seed germination potential of different transgenic plants under salt stress. **(F)** Quantification of % retained chlorophyll in the leaf disk of transgenic lines, VC and WT used for leaf senescence assay under 200 mM NaCl stress.
